# Quantity and Quality in Scientific Productivity: The Tilted Funnel Goes Bayesian

**DOI:** 10.3390/jintelligence10040095

**Published:** 2022-11-01

**Authors:** Boris Forthmann, Denis Dumas

**Affiliations:** 1 Institute of Psychology, University of Münster, 48149 Münster, Germany; 2 Department of Educational Psychology, University of Georgia, Athens, GA 30602, USA

**Keywords:** quantity, quality, equal odds baseline, creative scientific productivity, Bayesian modeling

## Abstract

The equal odds baseline model of creative scientific productivity proposes that the number of high-quality works depends linearly on the number of total works. In addition, the equal odds baseline implies that the percentage of high-quality works and total number of works are uncorrelated. The tilted funnel hypothesis proposes that the linear regression implied by the equal odds baseline is heteroscedastic with residual variance in the quality of work increasing as a function of quantity. The aim of the current research is to leverage Bayesian statistical modeling of the equal odds baseline. Previous work has examined the tilted funnel by means of frequentist quantile regression, but Bayesian quantile regression based on the asymmetric Laplace model allows for only one conditional quantile at a time. Hence, we propose additional Bayesian methods, including Poisson modeling to study conditional variance as a function of quantity. We use a classical small sample of eminent neurosurgeons, as well as the brms Bayesian R package, to accomplish this work. In addition, we provide open code and data to allow interested researchers to extend our work and utilize the proposed modeling alternatives.

## 1. Introduction

In order to make a meaningful impact in their field, should scientists focus on producing a large quantity of papers? Chance models of scientific productivity such as Sinatra et al.’s *Q* model (Janosov et al. 2020; Sinatra et al. 2016) or Simonton’s equal odds baseline (EOB; Simonton 1988, 2004, 2010) model imply exactly that. For example, the equal odds baseline proposes that every creative work a scientist produces has (on average) ‘equal odds’ of being a ‘hit’ and being recognized as high-quality by their peers. Therefore, the number of high-quality papers, is a linear function of the number of papers and the chance to produce a scientific ‘hit’ increases simply as a function of a researcher’s productivity.

Yet, it has been highlighted that the equal odds baseline also suggests other propositions and characteristics concerning the relationship between quantity and quality of ideas (Forthmann et al. 2020b, 2020c, 2021b). First, the correlation between the number of papers and average paper quality is proposed to be zero, such that individual scientists’ ‘hit-rate’ is not dependent on their quantity of work (Simonton 1988, 2003, 2004). Second, scientists that produce the highest quantity of work are also expected to vary the most from one another in terms of the overall quality of their output (Forthmann et al. 2021b). 

What this means in a linear regression model in which quantity predicts quality of production is that the equal odds baseline implies heteroscedasticity and model residuals are hypothesized to display the shape of a tilted funnel that increases across the scale of quantity (Forthmann et al. 2020c). In order to test the EOB in general and this ‘tilted funnel hypothesis’ of the EOB, statistical methods such as structural equation modeling (Forthmann et al. 2020b, 2021a) and quantile regression (Forthmann et al. 2020c) have been proposed and employed. The current work extends these previous efforts to test the EOB by transitioning to Bayesian statistical inference within a regression framework that encompasses and, hence, unifies existing approaches.

### 1.1. The Equal Odds Baseline and the Tilted Funnel Hypothesis

In general, research on creative productivity relies on open-ended count variables as measures of quantity of output (e.g., number of publications, number of musical compositions, number of responses in a divergent thinking test, and the number of generated ideas in a brainstorming session). Beyond quantity, each single product can also be evaluated for its creative quality. In its simplest variant, such an evaluation might lead to a dichotomous quality score (product is of high quality vs. product is not of high quality) and, in turn, a count of high-quality products. To add greater complexity, creative quality can be measured as an open-ended count variable, which has allowed for a more nuanced investigation into the correlates of creative quality. For instance, Simonton relied on the number of citations as the key quality indicator of a publication (e.g., Simonton 2003, 2004, 2009) and other researchers put forth similar arguments (e.g., Wang 2016). Citation counts can also be summed across publications to yield a quality score for an individual scientist’s overall output. All such quality scores in which scores of single products are summed across all products (e.g., all publications of a scientist) are referred to as additive quality scores (Forthmann et al. 2020a; Mouchiroud and Lubart 2001). Moreover, average creative quality scores (i.e., hit-ratios) result from dividing an additive quality score by the total number of products. Average quality scores are important for disentangling the intricate relationship between quantity and additive quality (Hocevar 1979; Forthmann et al. 2020a; Prathap 2018) and they allow the testing of competing explanations of the relationship between quality and quantity in creative production (Forthmann et al. 2020c; Kozbelt 2008).

The cross-sectional EOB is a parsimonious model for the relationship between additive quality *H* (e.g., the number of high-quality products) and quantity *T* (i.e., total production). The EOB posits that *H* is a linear function of individual differences in *T* (Simonton 2003, 2010):

(1) H=ρT+u

with regression slope ρ (i.e., hit-ratio) and random shock term *u* (Simonton 2004, 2009, 2010). The shock term in the model is incorporated to take individual differences in hit-ratios into account. Indeed, there is empirical evidence suggesting that some scientists produce a lot but are rarely cited (i.e., mass producers), whereas others publish rarely but almost exclusively high-impact works (i.e., perfectionists; Sidiropoulos et al. 2015; Cole and Cole 1967; Feist 1997). Consequently, in accordance with the EOB, detectable individual differences in hit-ratios beyond mere sampling variation are expected (Forthmann et al. 2020b, 2021b). Moreover, average quality *H*/*T* must be uncorrelated with *T* for the EOB to hold (Simonton 1988, 2003, 2004). If *H/T* were to be correlated with *T*, a non-linear relationship between *H* and *T* would be observed, therefore running counter to the basic linear tenet of the EOB (i.e., a quadratic term would be additionally needed in Equation (1) to predict *H* by *T*; Simonton 2003, 2004).

**Figure 1:**
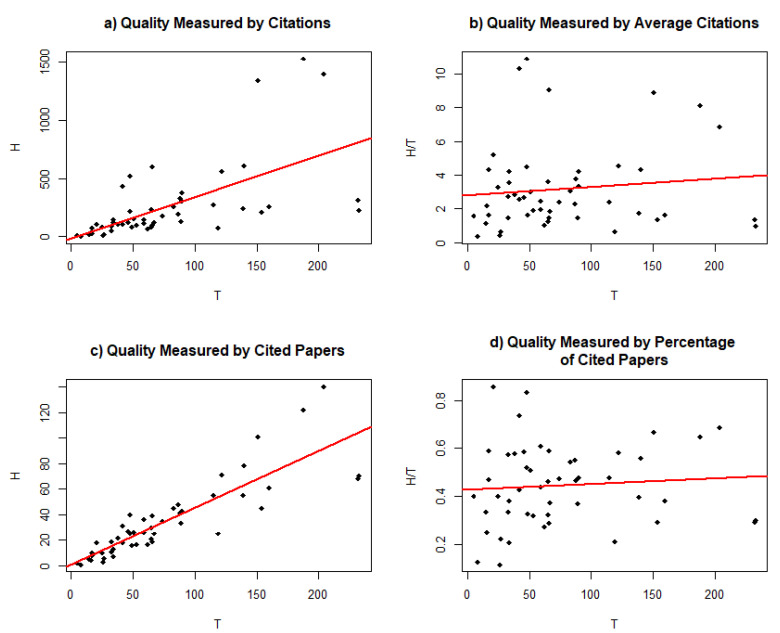
Graphical display of the data: (**a**) Relationship between number of citations (y-axis) and number of first-authored papers (x-axis); (**b**) relationship between average number of citations (y-axis) and number of first-authored papers (x-axis); (**c**) relationship between number of cited first-author papers and number of first-author papers; and (**d**) relationship between percentage of cited first-author papers (y-axis) and number of first-author papers. Red line depicts the ordinary least squares regression slope.

Another critical assumption related to the EOB has been coined the ‘tilted funnel hypothesis’ (Forthmann et al. 2020c). The tilted funnel hypothesis refers to the fact that residual variance—i.e., *Var*(*u*)—in the EOB cannot be homoscedastic. Instead, for lower values of *T*, residual variance must be lower than it would be for higher values of *T*. Hence, a bivariate scatterplot with *H* on the y-axis and *T* on the x-axis (cf. left sides in Figure 1) should give the impression of a funnel tilted to the right. Forthmann et al. (2020c) have examined this by means of quantile regression. In quantile regression, the tilted funnel hypothesis leads to the expectation that regression slopes at higher conditional quantiles of the distribution of *H* (e.g., the 0.80 quantile) are larger as compared to regression slopes at lower conditional quantiles (e.g., the 0.20 quantile), such that the additive quality of work steeply increases for every unit increase in quantity of work for those scientists that have the highest additive quality. Furthermore, Simonton (2010) argued that the distribution of *u* should be approximately log-normal. In this work we aim to estimate a model in which *u* is normally distributed on the log-scale (i.e., an overdispersed Poisson formulation of the EOB; for details see Section 2.1).

### 1.2. Aims of the Current Work

The aim of the current paper was to test approaches that aim to model the EOB within Bayesian regression frameworks. Importantly, Bayesian statistical analysis is increasingly used and discussed as a viable alternative to classical frequentist statistical approaches (especially when it comes to null hypothesis testing) in various scientific fields, such as psychological science (Wagenmakers et al. 2018) and scientometrics (Schneider 2018). The expected benefits of Bayesian modeling include greater flexibility (e.g., ways to account for heteroscedasticity) or availability of information for Bayesian inference. For example, Bayesian modeling provides complete sampling distributions for all estimated model parameters. Hence, even inference with respect to the random error term *u* can be facilitated this way. In addition, previous work in which the tilted funnel hypothesis was tested relied on quantile regression, which typically requires a different software package than other EOB modeling approaches (e.g., using structural equation modeling software packages). In this work, we employed Bayesian quantile regression (i.e., the tilted funnel goes Bayesian) and other EOB models within the same regression framework as implemented in the R package brms (Bürkner 2017, 2018). Thus, the overall steps of the analysis testing the propositions of the EOB can be carried out within a unified framework.

As a more far-reaching goal, this current study may pave the way for future EOB modeling when only small samples are available. When frequentist approaches are not available, rather complex models (i.e., in relation to sample size) can be fitted by means of Bayesian modeling, if weakly informative priors are used. This capability of Bayesian modeling is currently being discussed in relation to measurement invariance testing (van de Schoot et al. 2013), for example. In further support of this general aim of our work, we intentionally use a small classical sample of eminent neurosurgeons (drawn from Davis 1987) which allows for quick Bayesian model estimation. Other researchers, who are hopefully inspired by this work, can readily use the openly available data and code used in this work to test, refine, and extend Bayesian EOB modeling.

## 2. Methods

We reanalyzed a dataset of 50 American neurosurgeons who were active within the time period from 1965 to 1979 (Davis 1987). Notably, Simonton argued that the cross-sectional EOB is more likely to hold if ideas (i.e., those obtained by researchers during training and so forth) are sampled randomly from a domain (Simonton 2010). This would clearly be more likely when people are also randomly chosen. Yet, the sample of neurosurgeons used here was not randomly chosen as all of the people in the sample were eminent neurosurgeons (i.e., non-prolific neurosurgeons had no chance to enter the sample). The data reported by Davis were recovered by Forthmann et al. (2021b) who found that the dataset mostly adhered to the EOB tenets. In this work, we use this dataset for illustration and proof of concept of the proposed Bayesian modeling of the EOB. We explored the proposed models for two measures of quality: (a) total number of citations for papers; and (b) number of first-authored papers that received at least one citation. The measure of quantity was the number of first-authored papers. For exact details on how bibliometric data were retrieved, consult Davis (1987). Importantly, correlations between quantity and quality measures as reported in the papers (between quantity and citations: *r* = 0.62; between quantity and cited papers: *r* = 0.86) were almost exactly recovered (between quantity and citations: r = 0.61; between quantity and cited papers: r = 0.86; see Forthmann et al. 2021b). 

### 2.1. Bayesian Estimation

All models were estimated with the statistical software R (R Core Team 2021) by means of the brms package (Bürkner 2017, 2018) for Bayesian regression modeling which relies on the stan package for model estimation (Carpenter et al. 2017). All files needed to reproduce the reported analysis in this paper are openly available in a repository of the Open Science Framework (https://osf.io/yq5mb/).

The tilted funnel hypothesis was tested with Bayesian quantile regression (Yu and Moyeed 2001) which is implemented in brms via the asymmetric Laplace distribution. We estimated two models, one at the 0.20-quantile and one at the 0.80 quantile. The difference in the slope coefficients obtained from these models was derived from the difference in the posterior samples of the respective slopes. As we expected the slope at the 0.80-quantile to be higher than the slope at the 0.20-quantile (i.e., the tilted funnel hypothesis), we subtracted the slope of the 0.20-quantile from the slope of the 0.80-quantile such that a positive difference value is in accordance with the tilted funnel hypothesis. We further examined a Bayesian 95% credible interval of the slope difference.

Next, we fit an EOB model based on the normal distribution by omitting the intercept and using a constant prior for the slope parameter set to $\bar{H}/\bar{T}$ (Forthmann et al. 2021a). The same model was fit with the intercept and slope parameters freely estimated. We further fitted the EOB model as a simple linear regression, but the residual standard deviation was regressed on the number of publications in accordance with the tilted funnel hypothesis. We also tried to fit such a model with the residual standard deviation as a function of the number of publications and freely estimated intercept and regression slope, yet for this model the estimation process terminated with several technical issues flagged for both quality measures considered in this work. Then, the EOB was fit as a Poisson model. For this model, the intercept at the log-level was fixed to $\log\left(\bar{H}/\bar{T}\right)$ and the logarithm of the number of publications was added as an offset at the log-level, which implies

(2) $$H=\exp\left(\log\left(\bar{H}/\bar{T}\right)\right)\exp\left(\log\left(T\right)\right)=\rho T$$

Indeed, this model did not require any parameters to be estimated and was run with the argument algorithm set to “fixed_param”. For model comparison purposes, however, we fitted a variant of this model in which the slope was freely estimated. In a final model, we extended Equation (2) by explicitly adding the *u* parameter to the Poisson model as a random effect across authors (i.e., analogous to overdispersed Poisson modeling) resulting in

(3) $$H=\exp\left(\log\left(\bar{H}/\bar{T}\right)\right)\exp\left(\log\left(T\right)\right)\exp\left(\upsilon \right)=\rho Tu$$

with exp(υ) = *u* and $\upsilon \;\sim \;N\left(0,\;\sigma_{\upsilon }\right)$. In this model, again log-quantity was added as an offset and the log-level intercept was fixed to $\log\left(\bar{H}/\bar{T}\right)$.

All models were fit to both dependent variables and used the brms’ default priors when possible. However, the simple EOB model based on the normal distribution with citations as the dependent variable, and in which the residual standard deviation was modeled as a function of the number of first author papers, produced technical errors. Weakly informative priors were needed to fit this model. Given that this model could be estimated without technical problems for the number of cited first author papers, we rescaled the intercept and slope estimates obtained for the prediction of the residual standard deviation. These rescaled estimates were used as means in normal priors that had both a standard deviation of 0.25. With this setup, model estimation terminated without technical errors. All models were estimated with four chains and 2000 iterations. Only for the overdispersed Poisson variant of the EOB model, the number of iterations was increased to 5000. All available convergence diagnostics (i.e., $\hat{R}$, Bulk-ESS, and Tail-ESS; see (Vehtari et al. 2021)) were in the recommended ranges, which flagged that Bayesian inference was accurate. Next, the expected log-pointwise predictive density (ELPD; Vehtari et al. 2017) was used for model comparison purposes. Thus, models were compared in terms of their expected capability to predict new data. The best-fitting model has the highest ELPD and is used as a reference for evaluation of ELPD differences (i.e., the best-fitting model receives an ELPD difference of zero). Specifically, we used ELPD differences and respective standard errors for multi-model inference. We consider ratios of 2 of ELPD difference and the corresponding *SE* as substantial. Finally, we focus on estimates of *u* as a reflection of individual differences in the hit-ratio. We checked the relative positioning of neurosurgeons by correlational analysis of the *u* estimates derived from the various models.

## 3. Results

### 3.1. Citations as a Measure of Quality

As expected, the slope for the conditional 0.80-quantile was significantly larger when compared to the slope for the conditional 0.20-quantile (see Table 1, difference = 4.29, 95%-CI: [2.64, 6.20]). Hence, Bayesian quantile regression provided evidence for the tilted funnel hypothesis, which implies heteroscedastic residual variance and steeper regression slopes at higher quantiles of the quality distribution (see also Figure 1 top left).

The best fitting model, as per ELPD differences, was the overdispersed Poisson model (see Table 2). The EOB model based on the normal distribution and explicit modeling of the residual standard deviation as a function of quantity was the second-best model. However, when directly comparing this model with the third best model (i.e., the simple EOB model), the standard error of the ELPD difference was more than twice as large as the ELPD difference (ELPD difference = −9.20, *SE* = 20.60) suggesting that modeling heteroscedasticity does not substantially improve model fit beyond the simple EOB model. In addition, the simple EOB model and the simple regression model differed substantially in terms of ELPD difference (ELPD difference = −3.60, *SE* = 0.90). In accordance with previous analysis of this dataset, this provided evidence in favor of the EOB, which is also a more parsimonious model when compared to the simple linear regression model. The simple Poisson model was by no means competitive, with a very large ELPD difference to the overdispersed Poisson model (i.e., much larger when compared to any of the other models tested; see Table 2).

The correlational analysis (see lower Triangle in Table 3) demonstrated that only individual hit-rate deviations of the neurosurgeons for the overdispersed Poisson model provided somewhat different *u* estimates. The shared variance with the *u* parameters with any other estimate was approximately 66%, while all other correlations were indistinguishable from unity.

**Figure 2:**
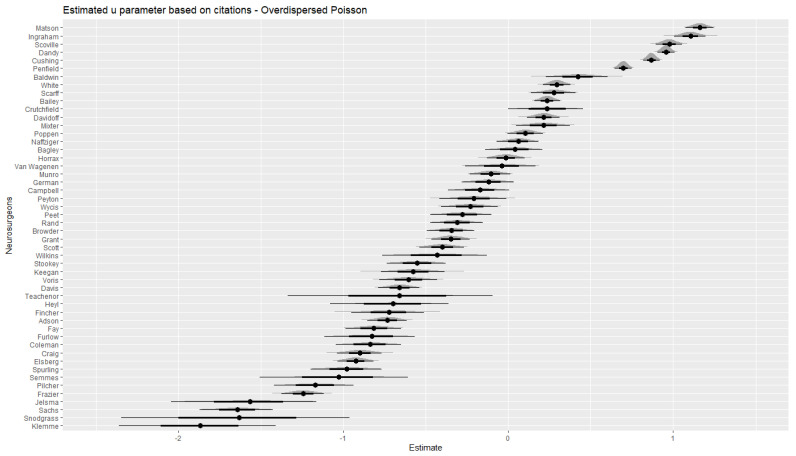
Estimated individual deviations from the overall hit rate obtained from the overdispersed Poisson model for citations.

In Figure 2, we display the *u* parameter estimates obtained from the overdispersed Poisson model along with credible intervals and densities of the respective sampling distributions. This highlights that the top-6 neurosurgeons with respect to the *u* parameter had significantly higher values when compared to the rest of the neurosurgeons. This is indicated by the fact that their credible intervals did not overlap with the credible intervals of all other neurosurgeons (admittedly, there was overlap between Penfield and Baldwin, but that was the only one). In addition, the dependence of measurement precision on the number of papers is clearly visible here. Those neurosurgeons with the smallest numbers of first author papers (e.g., Snodgrass or Teachenor) had the widest credible intervals (i.e., the lowest measurement precision), whereas the neurosurgeons with the largest numbers of first author papers (e.g., Elsberg, Frazier, or Penfield) tended to have narrower credible intervals. Yet, the number of citations also affected measurement precision as the neurosurgeons with comparably large efficiency (i.e., a large ratio of citations to publications) had the narrowest credible intervals (e.g., again the top-six illustrate this).

### 3.2. Number of Cited First Author Papers as a Measure of Quality

As expected, the slope for the conditional 0.80-quantile was significantly larger when compared to the slope for the conditional 0.20-quantile (see Table 1, difference = 0.33, 95%-CI: [0.24, 0.40]). Hence, across both examined dependent variables, evidence in favor of the tilted funnel hypothesis was found. Again, heteroscedastic residual variance provided a more realistic account for the data when the number of cited first author papers was the dependent variable (see also Figure 1 bottom left).

The model comparison findings for cited first-author papers (see Table 4) as the dependent variable replicated the findings for citations (cf. Table 2). Yet, the difference between the explicitly heteroscedastic EOB model and the simple EOB model was more substantial for the cited paper model (ELPD difference = −24.30, *SE* = 5.60). Similarly, the comparison between the simple EOB model and the linear regression model was substantial (ELPD difference = −4.00, *SE* = 1.20) and, hence, the EOB was a comparably better model than the more general linear regression.

Also, for cited papers as the quality measure, the *u* estimates obtained from the overdispersed Poisson model were the only ones that differed in notable ways from the other estimates (see the upper triangle in Table 3). The shared variance of the *u* estimates derived from the overdispersed Poisson models with any other variant was approximately 76%. Again, all other correlations were very close to unity.

**Figure 3:**
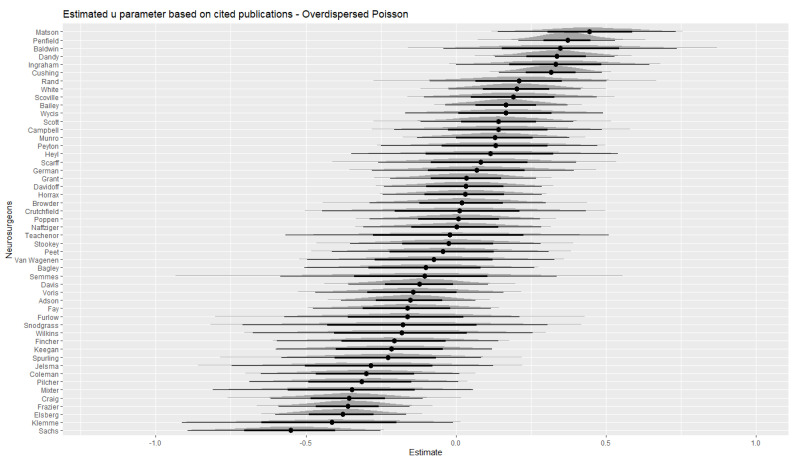
Estimated individual deviations from the overall hit rate obtained from the overdispersed Poisson model for cited papers.

In Figure 3, the *u* parameter estimates obtained from the overdispersed Poisson model are shown. Again, we also display the credible intervals and densities of the respective sampling distributions. Overall, the *u* estimates based on cited papers differentiated less strongly between neurosurgeons, which is highlighted by greater overlap between credible intervals. Thus, the number of cited papers as a measure of quality provided little diagnostic information in this regard in comparison to the total number of citations (cf. Figure 2).

### 3.3. Ranking the Most Creative Neurosurgeons

Table 5 shows the 10 most creative neurosurgeons as reported in Davis (1987) and how they ranked on the *u* estimates obtained in the current study. Eight out of ten of Davis’ top ten creative neurosurgeons were also among the top ten based on *u* parameter estimates based on the overdispersed Poisson model. The *u* parameter most likely reflects researcher capacity, institutional differences, luck (recall that the EOB is a chance model), and any other factors contributing to differences in individual impact here. Davis’s (1987) criteria for the most creative scientists were based basically on measures of *H* and *T* as proposed in the EOB and could be expected to be more unequivocal in comparison to the ranking in this study based on a parameter that is independent from the quantity-quality relationship. Hence, inspecting *u* parameter estimates adds to the information used by Davis (1987). This is even more emphasized here because the data adheres to the EOB. It was not clear beforehand that these ten eminent neurosurgeons also mostly had the highest hit rates. This is further highlighted by the fact that Davidoff and Elsberg did not have a top-10 *u* parameter. In particular, Elsberg is a notable case as he dropped to the very other end of the distribution of the set of fifty neurosurgeons. Based on the credible intervals depicted in Figure 2 and Figure 3, he clearly had a significantly lower *u* parameter as compared to the top-10. This difference between Elsberg and the others at the top-end of the distribution was much more pronounced for the citation model in comparison to the cited-papers model.

## 4. Discussion

In this work, we have shown how the assumptions underlying the EOB can be tested within a unified Bayesian regression modeling framework. First, we examined the tilted funnel hypothesis by means of Bayesian quantile regression (i.e., a regression model based on the asymmetric Laplace distribution). Second, we compared various formulations of the EOB model by means of Bayesian multi-model inference utilizing the ELPD difference. Like previous work using structural equation modeling frameworks such as those implemented in the R package lavaan (Rosseel 2012), we used the capabilities of brms to fix regression coefficients at their expected values under the assumption that the EOB model holds for the data. This model can be, and was, easily compared with a model in which intercept and slope are freely estimated. Across the dependent variables we tested here, we found that the EOB model with a fixed intercept and slope performed substantially better compared to the simple linear regression model with free estimates of coefficients. In addition, we found that a model that explicitly takes the tilted funnel hypothesis into account by regressing the residual standard deviation on quantity performed better when compared to the simple EOB model (yet not substantially better for the citation analysis). The best fitting model was an overdispersed Poisson model that included the *u* parameter as a normally distributed random effect at the log-level following the theorizing of Simonton (2010). Clearly, such a model is flexible enough to have the capability to handle the tilted funnel data pattern. A Poisson model without explicit modeling of the *u* parameter was by no means competitive.

Correlational analysis of residuals further showed that modeling choices have mostly negligible effects on the quantification of residuals. Only the *u* parameters obtained from the overdispersed Poisson displayed a visible degree of differentiation from the other model formulations. These observations are useful in case a researcher is interested in the *u* parameter as a quantification of individual differences in the hit-ratio. Conceptually, the *u* parameter refers to a researcher’s capacity to produce high-quality works, but also is theoretically inseparable from other factors such as luck, institutional factors (which are at least partially confounded with individuals in the EOB model), and other random sources (cf. Janosov et al. 2020; Sinatra et al. 2016). Simply speaking, the *u* parameter quantifies how a researcher performed in comparison to what was expected. Thus, in a sense, it quantifies a researcher’s efficiency and adds useful information beyond the strongly correlated indicators of *H* and *T*. This was illustrated by reconsidering Davis’ selected ten most creative neurosurgeons, which were chosen based on indicators of *H* and *T*, yet in the current work we observed that two of these eminent neurosurgeons were not in the top ten based on the *u* parameter estimates. While Davidoff dropped only a few ranking positions, Elsberg moved to the lower end of the distribution. Elsberg was clearly far less impactful than expected based on his level of productivity (i.e., he was a mass producer).

Thus, our empirical illustration emphasizes several advantages of Bayesian modeling. First, Bayesian modeling is very flexible. This is highlighted by modeling dispersion directly as a function of quantity in a heteroscedastic variant of the EOB, which was implemented within the model syntax of the brms package (Bürkner 2017, 2018). Second, there are other areas in which the capability of Bayesian modeling for small samples has been highlighted. For example, Bayesian modeling has been shown to be useful even for rather short single-case time series (Solomon and Forsberg 2017; Christ and Desjardins 2018). In this work, this is emphasized by the fact that the EOB model with explicit modeling of the tilted funnel pattern could not be estimated with the default priors implemented in brms. However, borrowing and rescaling the needed information from the same model successfully estimated for cited papers resulted in informative priors that allowed estimation of the model for citations. Third, sampling distributions for all model parameters are immediately available and provide critical information for Bayesian inference (this is not necessarily the case for frequentist approaches). We have illustrated this by looking more closely at the *u* parameter along with credible intervals. Credible intervals nicely show which *u* parameters were estimated with the least or with the highest measurement precision. Finally, it should be acknowledged that the practice of null hypothesis testing has been flagged by researchers as being logically flawed (e.g., Schneider 2018) and in this work we focused much more on multi-model inference rather than null hypothesis testing. Hence, looking at the EOB from a null hypothesis testing perspective is expected to further emphasize the usefulness and capability of Bayesian modeling to account for the quantity-quality relationship in scientific productivity.

It should be acknowledged that the sample size was very small, yet this was intentionally chosen. Besides its various advantages, sometimes Bayesian estimation can take weeks, and we found it constructive to examine ideas of Bayesian EOB modeling on a sample for which results are available within minutes. This allows other researchers to more quickly test and extend the analyses employed in this work. Finally, the way we derived credible intervals for the difference in slopes for different conditional quantiles was highly pragmatic. We argue that the reported differences, particularly the credible intervals for the difference, should be interpreted with caution. Simply subtracting the posterior samples carries the assumption that the sampling distributions of both slopes are independent. However, datasets that adhere to the EOB are more likely to produce sampling distributions that are positively correlated, which implies that statistical inference here was most likely conservative and trustworthy. We suggest that future work should investigate this in more detail.

## 5. Conclusions

We hope to have set the stage for Bayesian modeling of creative scientific productivity. The content and openly available material that is provided by this work will hopefully be a starting point for researchers to further experiment and extend the proposed approaches. Promising new avenues for research are multivariate extensions of the EOB that allow disentangling individual differences from some of the random factors inevitably included in the *u* parameter (see Caviggioli and Forthmann 2022). We expect that Bayesian modeling has great potential when such rather complex models are implemented, especially for smaller samples.

## Figures and Tables

**Table 1:** Model estimates for Bayesian quantile regression.

Model	Citations				Cited Papers			
Quantile	0.20		0.80		0.20		0.80	
	Estimate	95% CI	Estimate	95% CI	Estimate	95% CI	Estimate	95% CI
Population level								
$\beta_{0}$	2.54	[−30.73, 35.82]	−37.95	[−108.27, 34.99]	−0.36	[−3.42, 2.42]	−1.74	[−5.35, 2.58]
$\beta_{1}$	1.32	[0.85, 1.75]	5.61	[4.02, 7.44]	0.30	[0.27, 0.34]	0.63	[0.55, 0.70]
σ	38.70	[29.42, 52.02]	73.37	[54.93, 97.32]	3.12	[2.37, 4.16]	3.55	[2.69, 4.72]

Table notes. CI = credible interval.

**Table 2:** Model comparison results for citations as the dependent variable.

	Model				
	EOB Simple	EOB Heteroscedastic	Simple Regression	Poisson	Overdispersed Poisson
LOO comparison					
Model order	3	2	4	5	**1**
ELPD difference	−106.70	−97.50	−110.30	−3488.40	**0.00**
ELPD difference *SE*	8.60	21.50	9.10	824.20	**0.00**

Table notes. LOO = leave-one-out cross-validation. ELPD = expected log-pointwise predictive density. SE = standard error.

**Table 3:** Correlations between various *u* estimates.

	EOB Simple	EOB Heteroscedastic	Simple Regression	Poisson	Overdispersed Poisson
EOB simple	-	1.00	.99	.99	.87
EOB heteroscedastic	1.00	-	.99	.99	.87
Simple regression	.99	.99	-	.99	.87
Poisson	1.00	1.00	.99	-	.87
Overdispersed Poisson	.81	.81	.81	.81	-

Table notes. Lower triangle: Correlations between various *u* estimates for citations as the dependent variable. Upper triangle: Correlations between various *u* estimates for cited papers as the dependent variable.

**Table 4:** Model comparison results for cited papers as the dependent variable.

	Model				
	EOB Simple	EOB Heteroscedastic	Simple Regression	Poisson	Overdispersed Poisson
LOO comparison					
Model order	3	2	4	5	**1**
ELPD difference	−30.60	−6.20	−34.50	−51.90	**0.00**
ELPD difference *SE*	5.60	2.40	6.40	16.20	**0.00**

Table notes. LOO = leave-one-out cross-validation. ELPD = expected log-pointwise predictive density. SE = standard error.

**Table 5:** Rankings of the *u* estimates for the ten most creative neurosurgeons as per Davis (1987).

	Citations (cf. Figure 2)	Cited Papers (cf. Figure 3)
Baily	10	10
Cushing	5	6
Dandy	4	4
Davidoff	12	20
Elsberg	42	48
Ingraham	2	5
Matson	1	1
Penfield	6	2
Scoville	3	9
White	8	8

## Data Availability

All files to reproduce the reported analysis are available in a repository in the Open Science Framework: https://osf.io/yq5mb/.
